# Automated interpretation of time-lapse quantitative phase image by machine learning to study cellular dynamics during epithelial–mesenchymal transition

**DOI:** 10.1117/1.JBO.25.8.086502

**Published:** 2020-08-18

**Authors:** Lenka Strbkova, Brittany B. Carson, Theresa Vincent, Pavel Vesely, Radim Chmelik

**Affiliations:** a Brno University of Technology, Central European Institute of Technology, Brno, Czech Republic; b Uppsala University, Department of Immunology, Genetics, and Pathology (IGP), Rudbeck Laboratory, Uppsala, Sweden; c NYU School of Medicine, Department of Microbiology, New York, United States; d Brno University of Technology, Institute of Physical Engineering, Faculty of Mechanical Engineering, Brno, Czech Republic

**Keywords:** digital holographic microscopy, quantitative phase imaging, supervised machine learning, epithelial–mesenchymal transition

## Abstract

**Significance:** Machine learning is increasingly being applied to the classification of microscopic data. In order to detect some complex and dynamic cellular processes, time-resolved live-cell imaging might be necessary. Incorporating the temporal information into the classification process may allow for a better and more specific classification.

**Aim:** We propose a methodology for cell classification based on the time-lapse quantitative phase images (QPIs) gained by digital holographic microscopy (DHM) with the goal of increasing performance of classification of dynamic cellular processes.

**Approach:** The methodology was demonstrated by studying epithelial–mesenchymal transition (EMT) which entails major and distinct time-dependent morphological changes. The time-lapse QPIs of EMT were obtained over a 48-h period and specific novel features representing the dynamic cell behavior were extracted. The two distinct end-state phenotypes were classified by several supervised machine learning algorithms and the results were compared with the classification performed on single-time-point images.

**Results:** In comparison to the single-time-point approach, our data suggest the incorporation of temporal information into the classification of cell phenotypes during EMT improves performance by nearly 9% in terms of accuracy, and further indicate the potential of DHM to monitor cellular morphological changes.

**Conclusions:** Proposed approach based on the time-lapse images gained by DHM could improve the monitoring of live cell behavior in an automated fashion and could be further developed into a tool for high-throughput automated analysis of unique cell behavior.

## 1 Introduction

Currently, automated image acquisition systems enable microscopic experiments that generate large image datasets. Manual observation and evaluation of the microscopic images require a trained biologist who performs an inspection for every image, which is both time-consuming and requires considerable effort and concentration by the investigator. Human analysis can be biased, varying with skill and scientific rigor. Consequently, these and other aspects impose significant constraints on the speed, reliability, and validity of such evaluation of microscopic images.

One approach to address these limitations is supervised machine learning,^1^ which is increasingly being applied to the classification of microscopic data.^2^^,^^3^ As an objective unbiased method of scoring the content of microscopic images, this method has been argued to be more sensitive, consistent, and accurate in comparison to subjective manual interpretation.

When applying the supervised machine learning to cell classification, a computer is trained based on example images of cells belonging to predefined cell classes.^4^ Once segmented successfully, the cells are often represented by a set of unique features for the purpose of dimensionality reduction. The features are summarized into a feature vector, which serves as an input to the classifier. After the classifier is trained on the user-labeled training examples, it is then able to distinguish between a defined set of cell classes in an experimental sample.

When exploiting machine learning in light microscopy, most microscopic techniques only provide intensity images and do not detect the phase delay induced by the imaged cells. Digital holographic microscopy (DHM) enables such phase detection and hence provides quantitative phase images (QPIs) of live cells with high intrinsic contrast without labeling. The phase in the image corresponds to the dry mass density distribution within the cell and correspondingly it is quantitative in terms of cell mass. As such, DHM provides additional information, which has great potential for automated interpretation of cell behavior. Also other label-free microscopic techniques have been applied to cell imaging, including harmonic generation microscopy^5^^–^^7^ or Raman imaging.^8^ These methods, however, require intense laser light passing through the specimen that could influence the cell behavior and moreover entail scanning, hence are not widefield. Both approaches enable visualization of cell structure and function and could be considered complementary techniques to QPI.

Several studies have applied machine learning classification algorithms to QPI gained by DHM with outcomes such as morphology-based classification of red blood cells, automated detection and classification of living organisms in drinking water resources, and automated diagnosis of breast and prostate cancer from tissue biopsies.^9^^–^^12^ We have previously reported the advantage of an automated DHM-based analysis in the classification of different cell morphologies in response to nutritional deprivation. This study demonstrated that the quantitative nature of single-time-point images acquired by coherence-controlled holographic microscope (CCHM) improves the classification of cellular morphologies as compared to other techniques.^13^^,^^14^ However, some complex dynamic processes demand time-resolved live-cell imaging in order to gain more information and correctly interpret the cell behavior poststimuli. Efforts to analyze more complex dynamic cellular processes using single-cell kinetic states from holographic cytometry of human melanoma cells have also been reported, acquiring single-time-point images from time-lapse microscopy for analysis.^15^ Several other studies applied machine learning for classification of cells using time-lapse QPI.^16^^–^^18^

To our present knowledge, these studies have not applied machine learning for cell classification of time-lapse QPIs using the features extracted from time-lapse images, and thus a temporal context, for classification. We hypothesize that the inclusion of time data will allow better assessment and characterization of live cell behavior.

Herein we report a methodology for cell classification during cellular transitions based on time-lapse QPI using the Namru Mus musculus mammary gland (NMuMG) cell line, a well-established TGFβ-inducible epithelial–mesenchymal transition (EMT) model system.^19^^,^^20^ EMT is a fundamental process occurring during development and during pathological conditions, particularly in fibrosis and wound healing. In addition, EMT is believed to be the key, initial step in cancer metastasis and has been linked to chemotherapy resistance.^21^^,^^22^ During EMT, cell cycle is arrested, the cells lose their epithelial features and acquire a more mesenchymal, fibroblast-like phenotype visible as increased cell area and cell elongation.

Although EMT has been well characterized, a better understanding of the regulation and dynamics of this process is necessary to better predict disease progression and to develop novel therapies for metastatic disease. EMT in breast cancer cells has already been studied using QPI,^23^ but only single-time-point images were used for the monitoring. Given the gradual and time-dependent morphological changes occurring during EMT, assessment of only the epithelial and morphological end states will provide partial information about this complex transition. We therefore reasoned that analysis of EMT through time-lapse QPI may reveal novel cell phenotypic changes previously undetected. The additional benefit with using this microscopic approach is the lack of requirement for cell modifications with fluorescent reporters. The identification of novel physical characteristics of either end state, or of cells during this transition, could help provide guidance for additional studies and thus a better understanding of EMT and metastasis.

The two morphologically distinct phenotypes observed during EMT (epithelial and mesenchymal) represent the categories for cell classification in these sets of experiments. The time-lapse images of cells were obtained by CCHM^14^^,^^24^ during the 48-h TGFβ treatment period. The imaging in CCHM is based on the interference of the object and the reference light beams, which enables detection of the phase delay induced by the specimen. This quantitative nature of the images enables the extraction of features representing cell behavior, which formed input for the cell classification. The cells were classified by several supervised machine learning algorithms and the results were compared with the single-time-point approach used in our previous paper.^13^ Collectively, this system allowed assessment of the contribution of time-lapse QPI to cell classification of a dynamic and time-dependent cell phenotypic switch as well as comparison of this approach with classification based purely on single-time-point images.

## 2 Methods

### 2.1 Cell Culture Techniques

NMuMG cells (provided by Dr. Theresa Vincent) were grown in Dulbecco’s modified Eagle’s medium (Sigma-Aldrich, Czech Republic) supplemented with GlutaMAX™ (Life Technologies, Czech Republic), 10% fetal bovine serum (Sigma-Aldrich, Czech Republic), 100  U/ml penicillin, and 0.1  mg/ml streptomycin (Life Technologies, Czech Republic). The cells were harvested by trypsinization and transferred into 10 sterilized observation chambers μ-Slide I (Ibidi GmbH, Germany) at $50\;{\text{cells}/\mathrm{mm}}^{2}$ to ensure a low density for segmentation of individual cells. Once the cells were transferred to the observation chambers, they were kept in the incubator and imaged the day after. The 10 chambers were divided into control and TGFβ-treated (10  ng/ml) with imaging beginning immediately after treatment.

### 2.2 CCHM

**Fig. 1:**
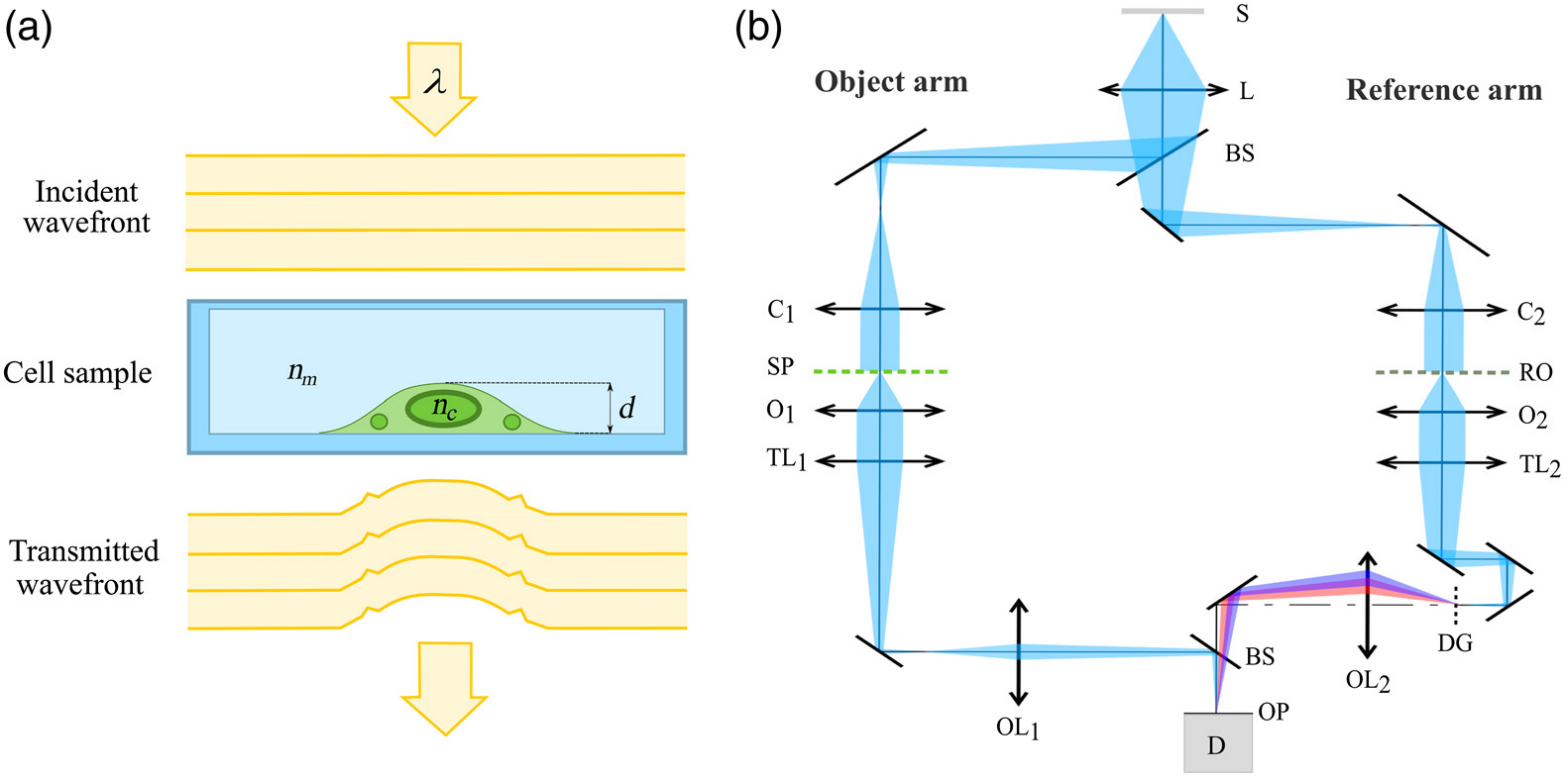
Imaging in CCHM. (a) Model of an adhered cell in the observation chamber imaged by CCHM. (b) Optical setup of CCHM: light source (S), relay lens (L), beamsplitters (BS), condensers (C), specimen (SP), reference object (RO), microobjectives (O), tube lenses (TL), diffraction grating (DG), output lenses (OL), output plane (OP), and detector (D).

Quantitative phase imaging of cells was performed by CCHM,^14^^,^^24^^,^^25^ now also available as Q-Phase (TESCAN ORSAY HOLDING, a.s., Brno, Czech Republic). The optical setup of the microscope is based on Mach–Zehnder type interferometer modified for incoherent, off-axis holographic microscopy as shown in Fig. 1(b). The illumination is formed by a low-coherence source (halogen lamp) while the beam is split into two separated optical pathways—reference and object arm. Both arms contain matching condensers, objectives, and tube lenses. In the reference arm, the diffraction grating is placed in order to ensure the achromatic formation of the interference pattern (hologram) in the output plane. The hologram is recorded by the CCD camera and numerically reconstructed using a Q–Phase software (TESCAN ORSAY HOLDING, a. s., Brno, Czech Republic). The numerical reconstruction of the image is based on carrier removal in the Fourier plane.^26^ The hologram is Fourier transformed using the 2-D fast Fourier transform (FFT) algorithm. The image spectrum in extracted by a windowing operation, whereas the window is centered at the carrier frequency. The frequency origin is translated to the center of the window, and the 2-D inverse FFT is applied to obtain the complex amplitude. Amplitude and phase are derived from the complex amplitude as modulus and argument, respectively. Since the values in the raw phase image are wrapped on the interval (−π,π), the phase unwrapping algorithm^27^ is applied. After the reconstruction, the image can still be burdened by the optical aberrations of the imaging system, imperfect adjustment of the microscope, or possibly by surrounding temperature changes. This issue is solved by the subtraction of the compensation surface described in detail in Ref. 28. In this way, a final unwrapped and compensated phase image is obtained. Such reconstructed QPI is proportional to the optical path difference of the two arms according to the following equation:

$$\varphi (x,y)=\frac{2\pi }{\lambda }d(x,y)[n_{c}(x,y)-n_{m}],$$ (1)

where λ is the illumination wavelength, d is the cell thickness, and $n_{c}$ is the mean axially integrated refractive index of the cell immersed in the culture medium of refractive index $n_{m}$^29^ as depicted in Fig. 1(a). According to Refs. 30 and 31, measured phase is proportional to the dry mass density within the cell (units of pg $\mu m^{-2}$), which can be obtained from the measured phase as follows:

$$\rho (x,y)=\frac{\lambda }{2\pi \gamma }\varphi (x,y),$$ (2)

where γ is the refraction increment.^29^ Based on the refractive index model of a cell introduced by Barer,^32^ the effective cell refractive index is linearly proportional to the concentration of protein in the cell where a proportionality constant is represented by γ. Several unconjugated proteins were measured, and the refraction increment for proteins is and approximated as 0.18 to 0.21  ml/g.

The use of incoherent illumination enables strong suppression of coherent noise and parasitic interferences. Moreover, the low illumination power of the source ($0.2\;\mu W/{\mathrm{cm}}^{2}$) is not likely to influence the physiological functions of imaged cells, making CCHM very convenient for live cell imaging.

### 2.3 Image Acquisition

The NMuMG cells were imaged by CCHM. During the experiment, the samples were illuminated with the halogen lamp through the interference filter (λ=650  nm, 10 nm FWHM). Microscope objectives (Nikon Plan Fluor 20×/0.5) were utilized for the imaging, providing the field of view 140  μm. For the purpose of classification, it was essential to acquire a reasonably large number of cells undergoing EMT. Therefore, six fields of view were imaged with a 5-min interval, each ∼1  mm apart from each other. Each chamber was imaged for 48 h in the presence or absence of TGFβ to obtain the time-lapse QPI for the classification. The media were not changed during the imaging period and conditions within the microscope mimicked that of the cell incubator (temperature 37°C) to ensure the cells were not subjected to stress.

**Fig. 2:**
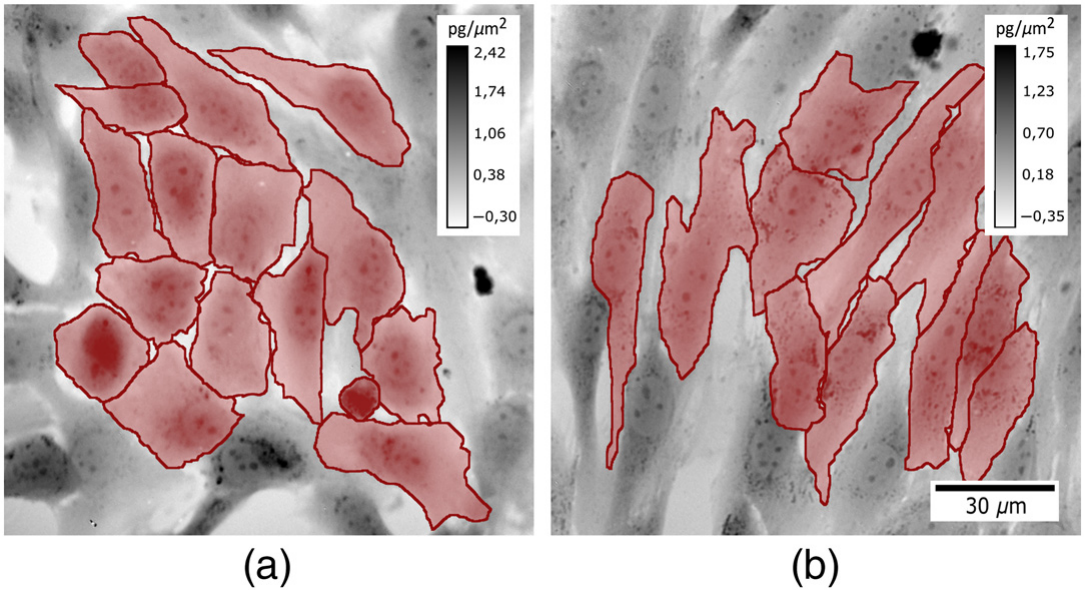
Examples of segmented QPIs of (a) epithelial and (b) mesenchymal phenotype gained by CCHM 2 and 30 h after the application of TGFβ, respectively. During EMT, the cell morphology changed from rounded to elongated, with the cell mass distributing relatively equally over the cell area, while the cell area increased significantly. QPIs are shown in grayscale in units of $pg/\mu m^{2}$ recalculated from phase (in radians) according to Davies.^31^

All time-lapse images of cells were gathered in the database. The database consisted of six 48-h-long records. Since none of the cells remained in the field of view for the whole imaging due to migration, 150 min (30 time-lapse images with interval 5 min) were determined as an optimal length of the time-lapse record for one cell. Hundred and eighty cells were chosen for the monitoring. Based on their morphology, the cells were labeled by the expert biologist as either epithelial (95 cells) or mesenchymal (85 cells). The cells with uncertain class membership were not considered and were excluded from the database. The two types of classified cell morphologies are shown in Fig. 2.

### 2.4 Classification

**Fig. 3:**
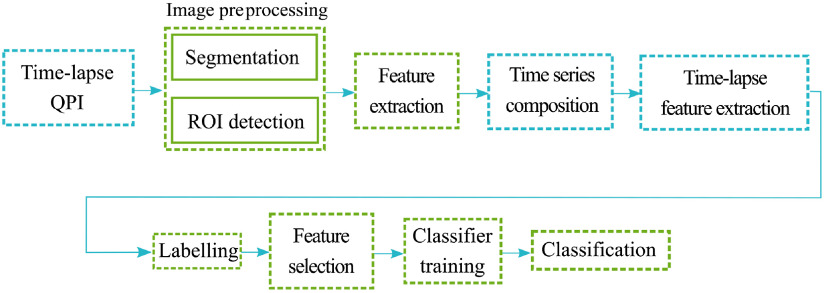
Overview of the proposed feature-based classification process based on time-lapse QPI. First, image preprocessing is carried out. The cells in the image are segmented from the background and identified as ROIs. Cell features are extracted for every ROI. Feature values in several time instants create a time series. Dynamic features are extracted from the time-series while creating the feature vectors representing behavior of cells. The data are split into training and testing set. Both training and testing data are labeled by the expert biologist. After the feature selection, testing data form input for the classifier. The classifier is trained on the training data and prepared to perform the classification on testing data.

Classification, as a category of supervised machine learning, aims to build a model that makes predictions based on self-learning procedure on known labeled data. In the case of cell classification, the algorithm identifies patterns in the input images and trains a model based on labels assigned to the cells by an expert biologist. Such trained model is able to classify cells in new, previously unseen images. The essential requirement for successful classification is a sufficiently large database of labeled cell images, in which the classifier is trained. The overview of the classification process based on time-lapse QPI is shown in Fig. 3 and is described in more detail in the following paragraphs.

### 2.5 Image Preprocessing and Feature Extraction

Before the classification itself, the cells were first segmented from the background in the time-lapse images by marker-controlled watershed segmentation approach.^33^ The individual cells were tracked using the cell tracking algorithm scripted in MATLAB (MathWorks, Inc.). The algorithm performs cell tracking by linking every segmented cell in the given frame to the nearest cell in the next frame. Only cells remaining in the field of view throughout the specified time were considered for assessment. Further, highly overlapping cells where the segmentation was not clear were not included, nor were cells located on the border of the image. The included cells were identified as separate regions of interest (ROIs), where each ROI was represented by a set of cell features—a procedure referred to as feature extraction. Two types of features were extracted from each cell: morphometric and QPI features. Morphometric features mostly reflect the shape of the cell. These features involved footprint area, perimeter of the footprint area, convex area, perimeter of the convex area, solidity, roundness, indentation, eccentricity, extent, and centroid of the cell. QPI features are extracted from the phase values of the cell in QPI and therefore, contain quantitative information about the dry mass density distribution within the cell. QPI features were composed of the total phase of the cell, average phase, median, variance, standard deviation, skewness, and kurtosis of the phase values. Both types of features are described in more detail in our previous work.^13^ In the next step, all extracted features undergo normalization in order to scale the feature values to a fixed range from 0 to 1.

Each cell in a time instant therefore is represented by a feature vector composed of the cell features captured. Since every cell was recorded in time, each cell feature provides a univariate time series composed of the values of cell features over time. Accordingly, the consideration of all cell features, gives rise to a multivariate time series. There are two possible ways for the representation of time series. In the first, the values of time series itself represent the input for the classification, which will be referred to as a value-based approach. In the second, the feature-based approach, the time series is further represented by the newly defined time-lapse features, which subsequently form the time-lapse feature vector.

**Fig. 4:**
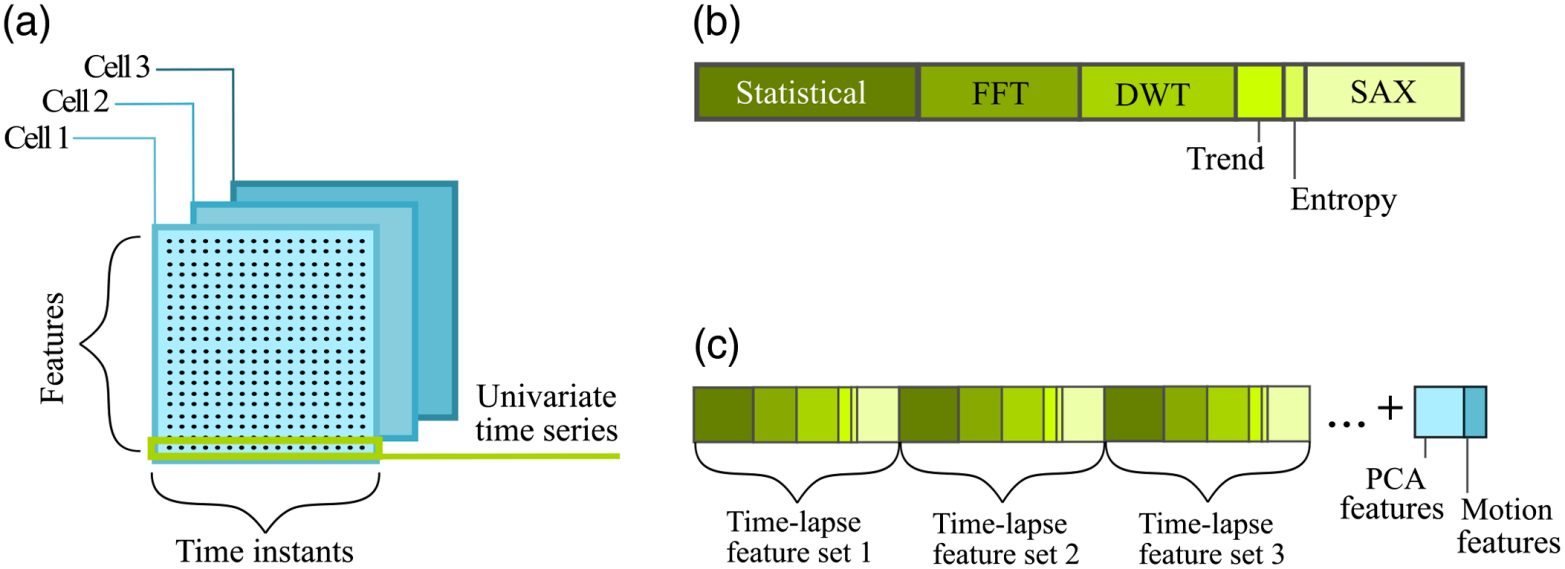
Overview of the time-lapse feature extraction process. (a) Each cell is represented by the multivariate time series composed of univariate time series (formed by cell feature values obtained within the time period). (b) Time-lapse feature extraction from univariate time series composing a partial time-lapse feature set. Individual segments represent the group of time-lapse features obtained by the extraction technique. The length of the segments indicates the approximate number of extracted time-lapse features for the group. (c) Construction of the final time-lapse feature vector. The final vector representing a single cell is formed by the concatenation of partial time-lapse feature sets belonging to a cell. In addition, the motion and PCA features are added.

In order to explain the formation of the final time-lapse feature vector in the feature-based approach, the brief notation will be introduced. Let $X=\{X_{1},X_{2},\ldots ,X_{Q}\}$ represent a collection of Q multivariate time series, where Q is the number of cells in the experiment. Each multivariate time series $X_{i}$ is formed by n observations (n is the number of time points) and d-dimensional variable (d is the number of cell features) as shown in Fig. 4(a). The multivariate time series $X_{i}$ can be written as

$$X_{i}=\{X_{\mathrm{ijt}}\},\;\text{for }j=1,\ldots ,d;t=1,\ldots ,n,$$ (3)

with the total number of observations d×n×Q.

We will consider the j’th component of the i’th time series $X_{ij}=\{X_{ij1},\ldots ,X_{ijn}\}$ to be a univariate time series. Therefore, the univariate time series will be composed of the values of one cell feature recorded in time. For each univariate time series $X_{ij}$, a feat time-lapse feature vector $M=(m_{1},m_{2},\ldots ,m_{L})$ is formed, where each m is a time-lapse feature extracted from the time series and L is the number of time-lapse features. In this way, each time series $X_{ij}$ is transformed into a partial time-lapse feature vector $M_{ij}$*.* Each multivariate time series is therefore transformed into d time-lapse feature vectors. The vectors are then concatenated into a final time-lapse feature vector of d×L dimensions.

The idea of the transformation is extracting information which would otherwise not be obvious as well as reducing the number of features compared to the value-based approach and therefore lowering computational time. The latter is obvious when encountering longer time series. There are several possible methods used for dealing with the feature-based representation of the time series. The employed feature extraction techniques are briefly described in the following paragraph.

Statistical features carry global information about the time series. The following metrics were chosen in order to statistically represent the structure of the time series: mean value, median value, standard deviation, minimum value, maximum value, skewness, and kurtosis. Fourier transform features are formed by the most significant coefficients gained by FFT algorithm. Wavelet transformation features are formed by detail and approximation coefficients computed by discrete wavelet transform algorithm. The trend is represented by the coefficients obtained by the linear least squares fitting of the time series and characterizes a long-term change in the mean value of a cell feature. Approximate entropy quantifies the unpredictability of fluctuations in the time series. The presence of repetitive patterns of fluctuation in a time series renders it more predictable and leads to a relatively small approximate entropy. Symbolic aggregate approximation (SAX) features are gained by SAX method,^34^ which is composed of two steps: piecewise aggregate approximation (PAA)^35^ and the conversion of a PAA sequence into a string composed of letters, where the original time series is converted to a symbol string.

All of the above time-lapse features were extracted from each of the univariate time series and created a partial time-lapse feature vector as shown in Fig. 4(b). Subsequently, the partial time-lapse feature vectors obtained from each univariate time series were concatenated into a final time-lapse feature vector, whereas other extracted time-lapse features (principal components analysis and motion features) were added on the tail as shown in Fig. 4(c).

Principal components analysis (PCA) features were gained by applying PCA^36^ on the whole multivariate time series, while mapping the multivariate data into a lower dimensional space. Motion features are composed of accumulated distance (overall distance travelled by the cell between the initial and the end point during the time interval), Euclidean distance (length of the straight line between the cell’s starting and end point reached during the time of monitoring), velocity (overall distance travelled by the cell over the elapsed time), and directionality (ratio of the Euclidian and accumulated distance). The position of the cell was determined by the cell centroids for all calculated motion features. Both PCA and motion features were added into the final time-lapse feature vector.

In the value-based approach, the extraction of time-lapse features is omitted, since the final time-lapse feature vector is composed of the raw data (values in each time point) contained in the multivariate time series. The final time-lapse feature vector is created by concatenating the univariate time series behind each other.

**Fig. 5:**
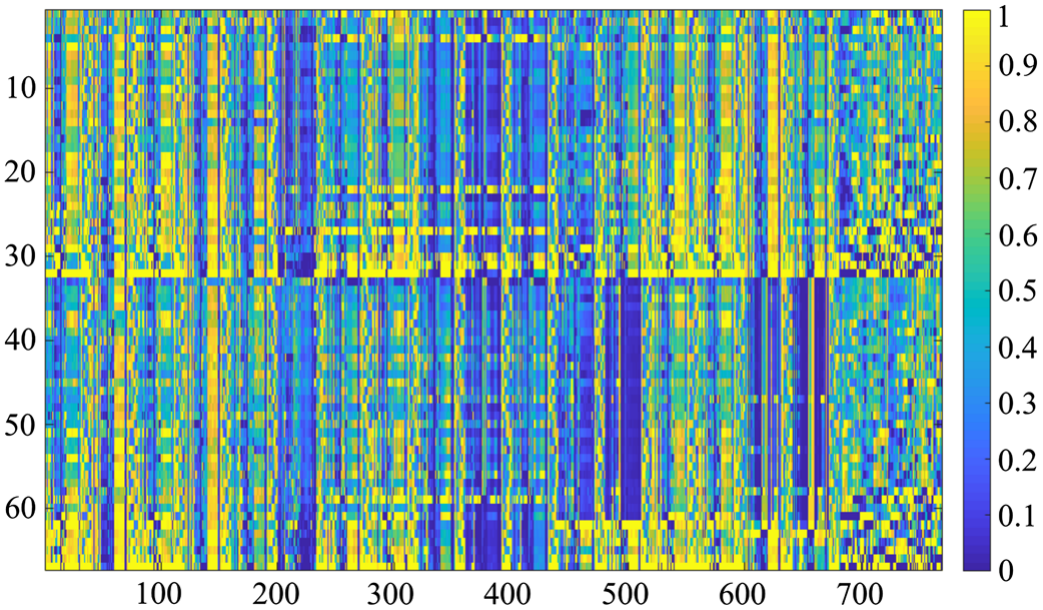
Example of the final time-lapse feature vectors concatenated into matrix. Elements of the matrix contain the (normalized) time-lapse feature values and are visualized using color: from blue (low values) to yellow (high values). First 32 rows represent time-lapse feature vectors extracted from epithelial cells and the other 35 rows from mesenchymal cells.

In both approaches, the final time-lapse feature vector represents a unique behavioral pattern of a cell. Before passing the vectors to the classification algorithms, the time-lapse feature values are scaled to a fixed range from 0 to 1. The example of a set of final time-lapse feature vectors gained by feature-based approach can be seen in Fig. 5, where the first 32 rows represent feature vectors extracted from epithelial cells and the other 35 rows from mesenchymal cells with the columns representing individual time-lapse feature values. The data are further split into training and testing set and are labeled by the expert biologist. Since the final time-lapse feature vectors are of substantial size, the next step is the selection of features with the highest potential to distinguish between the given classes, which would then form input for the machine learning classification algorithms.

### 2.6 Feature Selection

Since the time-lapse feature vectors are composed of a high number of features, feature selection is performed in order to reduce the dimensionality of the data, which leads to lower computation complexity and makes the training of the classification algorithms less time-consuming. Moreover, in this case, when the number of observations is limited in comparison to the large number of features, the limited observations may lead the learning algorithm to overfit to the noise. Reducing the number of features is therefore, in this case, an essential step before the classification.

We applied the filter approach for the feature selection.^37^ First, the t-test was applied to each feature and the p-value for each feature was compared as a measure of the feature’s ability to discriminate between the two classes. To estimate the order of class separation by the features, the empirical cumulative distribution function (CDF) of the p-values was computed. There were ∼15% of features, which have the p-values close to zero and 30% of features having the p-values smaller than 0.05. It can be concluded that there are roughly 200 features in the original time-lapse feature set, which have the potential to separate the two cell classes. In the value-based approach, there are ∼18% of features, which have the p-values close to zero and 30% of features having the p-values smaller than 0.05. CDF of the p-values showed that there are roughly 150 features from the original time-lapse feature set having rather high discriminative power.

**Fig. 6:**
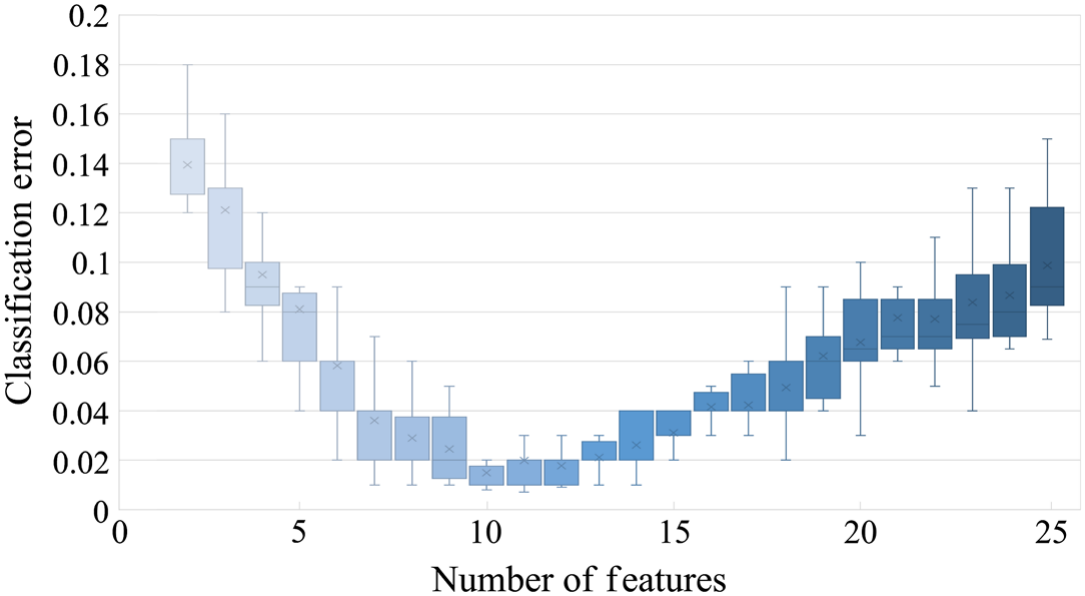
Classification error as a function of the number of features in feature-based approach.

The features were subsequently ordered by their p-values. In order to define the appropriate number of features to be selected, the classification error (the number of misclassified observations divided by the number of observations) as a function of the number of features was plotted. To obtain the classification error, several classification algorithms were employed. The results of the classification error in feature-based approach is shown in Fig. 6. The classification error was computed for different numbers of features between 2 and 25. The final number of selected features was determined as the mean value of the results produced by employing different classification algorithms. In the feature-based approach, the filter feature selection method obtains the smallest classification error when 10 features are engaged. Only these 10 features with the highest discriminative power are kept in the reduced time-lapse feature vectors used for the classification. In the value-based approach, 12 features were determined as optimal.

### 2.7 Classification Algorithms

After the features with the highest potential to distinguish between the epithelial and mesenchymal cell classes were selected, they create the input for the classification algorithms. Since the performance of the classification is highly dependent on the selection of the classification algorithm, we employed several supervised machine learning algorithms to correctly compare the performance of the classification based on single-time-point and time-lapse QPI. The following algorithms were tested in several possible variations with differently set parameters: decision trees (complex, medium, and simple tree, with defined maximum number of splits 100, 20, and 4, respectively), discriminant analysis (linear and quadratic discriminant), support vector machines (with linear, quadratic, cubic, and Gaussian kernel), k-nearest neighbor classifier (KNN), (fine KNN with k=1 and Euclidean distance, medium KNN with k=10 and Euclidean distance, cosine KNN with k=10 and cosine distance, cubic KNN with k=10 and cubic distance, weighted KNN with k=10, and weighted by the inverse square of the Euclidean distance) ensemble classifiers (bagged trees, boosted trees, subspace discriminant, and subspace KNN) and artificial neural network (feed-forward backpropagation neural network with one hidden layer containing 10 hidden neurons).

Several performance measures were calculated from the confusion matrix for each classification algorithm: accuracy, precision, recall, and F-score. Classification accuracy of a classifier is calculated as the ratio of the sum of the principal diagonal values to the sum of all values in the confusion matrix. It expresses the ratio of correctly classified examples by the classifier. Precision is the ratio of correctly classified positive examples to the total number of positive examples, while recall is the ratio of correctly classified positive examples to the all examples in actual class. F-score can be interpreted as a harmonic mean of precision and recall. Fivefold cross validation was used to evaluate the performance of the classification algorithms. The data were partitioned into five randomly chosen subsets of roughly equal size. One subset (testing set) was used for testing of the classifier, which had been trained on the remaining subsets (training set). This process was repeated five times, such that each subset was used for the validation. Since cross validation does not use all the data for training, it is a commonly used method to avoid overfitting. The overall performance of the classification algorithm was determined as the mean of performance measure values reached in the iterations. The whole classification procedure was performed in MATLAB.

## 3 Results and Discussion

After 48 h, the cells in the control conditions maintained their epithelial state, whereas the cells treated with TGFβ transitioned to the mesenchymal state as previously shown.^19^^,^^20^ We observed changes in cell morphology to occur ∼17  h posttreatment. The cells lost their epithelial features and acquired more mesenchymal, fibroblast-like phenotype. The cells became elongated, with the cell mass distributing relatively equally over the cell area, while the cell area increased significantly.

The classification was first performed on the reduced time-lapse feature vectors gained by value-based approach. The same procedure was then repeated for the reduced time-lapse feature vectors gained by feature-based approach. The classification was also performed on the features extracted from the single-time-point QPI to evaluate the contribution of the methodology based on time-lapse QPI.

The performance of the classification implementing the value-based approach is summarized in Table 1. The overall accuracy of the classification was 0.923±0.053. The overall precision, recall, and F-score were 0.907±0.052, 0.882±0.089, and 0.893±0.070, respectively. The performance of the classification using the feature-based approach is summarized in Table 2. Assessing the cell behavior by the time-lapse features led to higher performance of the classifier, as observed with the value-based approach, with the overall accuracy of the classification reaching 0.978±0.011. Further, with the incorporation of time-lapse features, the overall precision, recall, and F-score were 0.968±0.014, 0.961±0.013, and 0.964±0.013, respectively.

**Table 1:** Performance of the classification by supervised machine learning algorithms using value-based approach.

	Accuracy	Precision	Recall	F-score
Decision trees (complex)	0.79	0.84	0.631	0.726
Decision trees (medium)	0.876	0.836	0.826	0.831
Decision trees (simple)	0.889	0.863	0.829	0.842
Linear discriminant analysis	0.974	0.952	0.951	0.954
Quadratic discriminant analysis	0.919	0.89	0.882	0.893
SVM (linear)	0.951	0.943	0.928	0.932
SVM (quadratic)	0.971	0.963	0.947	0.953
SVM (cubic)	0.982	0.979	0.971	0.972
SVM (Gaussian medium)	0.98	0.984	0.981	0.983
KNN (fine)	0.945	0.941	0.926	0.93
KNN (medium)	0.918	0.896	0.887	0.891
KNN (cosine)	0.938	0.889	0.874	0.88
KNN (cubic)	0.889	0.852	0.84	0.844
KNN (weighted)	0.884	0.83	0.829	0.829
Bagged trees	0.82	0.827	0.705	0.761
Subspace discriminant	0.954	0.941	0.945	0.937
Subspace KNN	0.981	0.962	0.961	0.963
Boosted trees	0.931	0.913	0.906	0.909
Neural networks	0.952	0.941	0.942	0.943
Mean ± SD	0.923±0.053	0.907±0.052	0.882±0.089	0.893±0.070

**Table 2:** Performance of the classification by supervised machine learning algorithms using feature-based approach.

	Accuracy	Precision	Recall	F-score
Decision trees (complex)	0.981	0.969	0.962	0.965
Decision trees (medium)	0.986	0.981	0.975	0.976
Decision trees (simple)	0.991	0.983	0.981	0.981
Linear discriminant analysis	0.965	0.956	0.954	0.957
Quadratic discriminant analysis	0.981	0.969	0.959	0.961
SVM (linear)	0.951	0.952	0.944	0.947
SVM (quadratic)	0.989	0.986	0.981	0.985
SVM (cubic)	0.991	0.984	0.971	0.982
SVM (Gaussian medium)	0.991	0.989	0.98	0.981
KNN (fine)	0.971	0.961	0.945	0.952
KNN (medium)	0.988	0.981	0.962	0.97
KNN (cosine)	0.982	0.977	0.963	0.971
KNN (cubic)	0.965	0.955	0.951	0.95
KNN (weighted)	0.974	0.951	0.948	0.949
Bagged trees	0.987	0.97	0.971	0.973
Subspace discriminant	0.981	0.965	0.956	0.958
Subspace KNN	0.964	0.941	0.942	0.941
Boosted trees	0.969	0.954	0.949	0.952
Neural networks	0.973	0.962	0.956	0.958
Mean±SD	0.978±0.011	0.968±0.014	0.961±0.013	0.964±0.013

In order to correctly evaluate the benefit of incorporating the temporal information over the classification based solely on the static QPI, the classification was performed also on the static QPIs of cells undergoing EMT. The static QPI images were obtained from the time-lapse data by selecting one image from each time-lapse sequence. The classification of epithelial and mesenchymal cells based on the static QPI was performed according to the methodology previously described in our paper.^13^ The performance of the classification based on single-time-point QPI is summarized in Table 3. The overall accuracy of the classification was 0.889±0.053. The overall precision, recall, and F-score were 0.873±0.053, 0.838±0.198, and 0.853±0.077, respectively.

**Table 3:** Performance of the classification by supervised machine learning algorithms using the static QPI.

	Accuracy	Precision	Recall	F-score
Decision trees (complex)	0.891	0.839	0.833	0.83
Decision trees (medium)	0.886	0.86	0.882	0.872
Decision trees (simple)	0.923	0.929	0.918	0.922
Linear discriminant analysis	0.962	0.946	0.945	0.945
Quadratic discriminant analysis	0.892	0.842	0.82	0.832
SVM (linear)	0.912	0.897	0.879	0.888
SVM (quadratic)	0.88	0.961	0.949	0.955
SVM (cubic)	0.944	0.934	0.927	0.93
SVM (Gaussian medium)	0.937	0.912	0.906	0.907
KNN (fine)	0.895	0.869	0.844	0.857
KNN (medium)	0.762	0.808	0.618	0.701
KNN (cosine)	0.899	0.867	0.85	0.857
KNN (cubic)	0.79	0.791	0.632	0.697
KNN (weighted)	0.791	0.763	0.661	0.71
Bagged trees	0.871	0.841	0.775	0.806
Subspace discriminant	0.89	0.849	0.837	0.842
Subspace KNN	0.94	0.919	0.895	0.908
Boosted trees	0.935	0.917	0.91	0.914
Neural networks	0.89	0.846	0.842	0.841
Mean ± SD	0.889±0.053	0.873±0.053	0.838±0.098	0.853±0.077

**Fig. 7:**
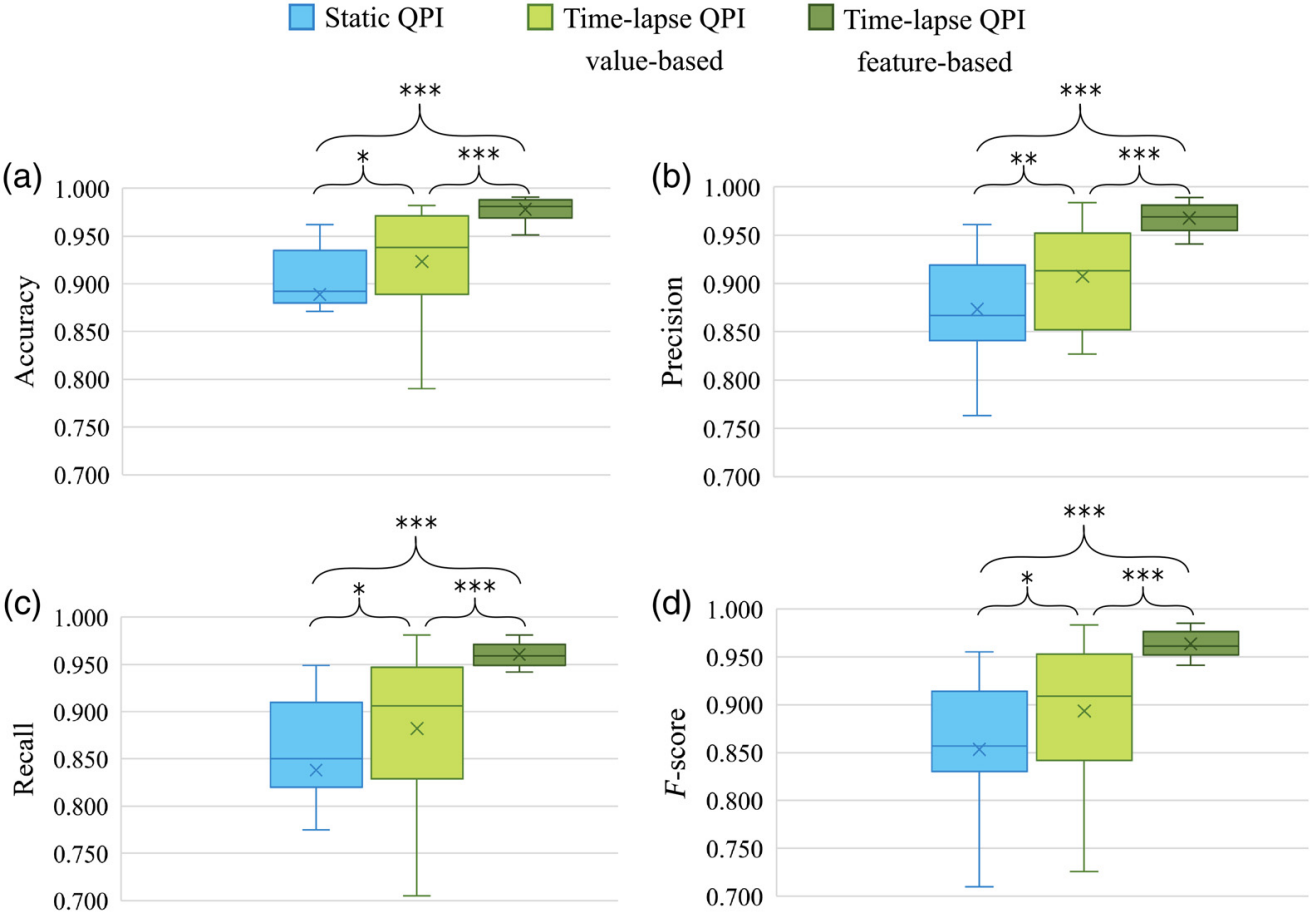
Box-whisker plots of overall classification performance of classification based on static QPI, time-lapse QPI (value-based and feature-based approach): (a) accuracy, (b) precision, (c) recall, and (d) F-score. Wilcoxon signed rank test was used for the statistical analysis. Symbols indicating significance are placed above (*p<0.05, **p<0.01, and ***p<0.001).

The performance of the classification obtained by the mentioned classification approaches was compared by statistical hypothesis testing. The Wilcoxon signed rank test was used in order to reveal the significant differences between the three distributions, with a null hypothesis that the median difference between pairs of observations is zero and a p-value of 0.05 to be considered statistically significant. The test revealed very significant differences between the feature-based and value-based time-lapse classification approaches (p<0.001) in terms of all performance parameters (accuracy, precision, recall, and F-score). Significantly different results (p<0.001) were obtained also from the classification based on static QPI and the classification based on time-lapse QPI employing the feature-based approach. According to the test, the classification based on static QPI and the classification based on time-lapse QPI using the value-based approach provided different performance of the classification with a lower significance (p<0.01 for precision and p<0.05 for other performance parameters). The performance results of all approaches are shown in the form of box-whisker plots in Fig. 7.

Several conclusions can be drawn from the results of the classification. The classification based on time-lapse QPI using either value-based or feature-based approach outperforms the classification based on static QPI, which does not consider the temporal information. Hence, taking into account the time information appears to improve the classification of the two cell phenotypes by nearly 9% in terms of accuracy. However, when it comes to the classification based on time-lapse QPI, the feature-based approach outperforms the value-based approach. The low-performance values in the value-based approach can be due to many factors, but mainly a consequence of the features, which are in this case the raw time series data, not fully representing the cell behavior. The other possibility is the increased sensitivity of this approach to the amount of noise in the time series.

Although the performance of the classification based on time-lapse QPI using the feature-based approach was relatively high, further improvement could be achieved by enlargement of the time-lapse QPI dataset, which would allow the classification algorithms to improve the training based on more extensive data.

Continued QPI-based classification of cell phenotype during EMT, beyond the epithelial and mesenchymal states, may allow for further understanding of this cell identity switch and thus cancer progression. Accordingly, even though the methodology was assessed using analysis of one specific cellular process, we suggest this analysis will be informative in the study of other dynamic cellular phenomena such as monitoring of cell cycle progression, cell death, and cellular response to external stimuli.

## 4 Conclusions

We have proposed a new methodology for cell classification based on the time-lapse QPIs using cells undergoing EMT as the biological system of focus. We have applied several supervised classification algorithms to differentiate between two distinct cell morphologies. Our findings show that the extraction of the time-lapse features representing dynamic cell behavior outperforms analysis based solely on the single-time-point QPIs, which indicates the importance of incorporating temporal information into the classification process. Despite the challenging time-lapse feature extraction, the proposed approach provides a novel, yet efficient way to classify the cells in QPIs with promising performance results. This approach could improve the monitoring of live cell behavior in an automated fashion and we believe that exploiting the methodology in QPI could contribute to promoting the DHM as an analysis tool and potentially a standard diagnostic technique used in biology and medicine.
